# RAS mutation status and immune microenvironment define distinct prognostic landscapes and predict chemotherapy benefit in pMMR colorectal cancer

**DOI:** 10.3389/fimmu.2026.1798858

**Published:** 2026-05-07

**Authors:** Shigang Wu, Jinbang Li, Zhe Chen, Jihong Liu, Feng Luo, Bingquan Li, Jun Zeng, Kunping Liu

**Affiliations:** 1 Department of Pathology, The Affiliated Qingyuan Hospital (Qingyuan People’s Hospital), Guangzhou Medical University, Qingyuan, Guangdong, China; 2 Department of General Surgery, The Affiliated Qingyuan Hospital (Qingyuan People’s Hospital), Guangzhou Medical University, Qingyuan, Guangdong, China

**Keywords:** CD163, colorectal cancer, immune microenvironment, prognosis, RAS

## Abstract

**Purpose:**

The aim of this study was to define the interplay between K/N-RAS mutational status, tumor immune microenvironment (TIME), and chemotherapy response in patients with proficient mismatch repair (pMMR) colorectal cancer (CRC)—a population refractory to immunotherapy but constituting >90% of CRC cases.

**Methods:**

We retrospectively analyzed 225 patients with pMMR CRC. Multiplex immunohistochemistry was used to quantify stromal and intraepithelial densities of CD4^+^, CD8^+^, CD68^+^, and CD163^+^ immune cells, along with expression of β-catenin, CMTM6, and programmed death-ligand 1 (PD-L1). Multivariable Cox regression and interaction tests were performed to identify prognostic and predictive biomarkers.

**Results:**

K/N-RAS status stratified pMMR CRC into two distinct subtypes. In RAS wild-type tumors, high stromal CD4^+^ T-cell infiltration was independently associated with better prognosis. In RAS-mutant tumors, loss of intraepithelial CD8^+^ T cells and high CD163^+^ macrophage density predicted poor survival. Critically, chemotherapy significantly improved overall survival only in specific subgroups, including RAS-mutant, β-catenin-low, CMTM6-low, and CD163-high patients. Formal interaction testing confirmed CD163^+^ macrophage infiltration as a predictive biomarker for chemotherapy benefit and significant chemotherapy benefit in the CD163-high subgroup (HR = 0.20, *p* < 0.001).

**Conclusions:**

RAS mutational status shapes a divergent immune landscape that dictates both prognosis and chemotherapy response in pMMR CRC. Integration of RAS status with key TIME features—particularly CD163—may enable the precise selection of patients most likely to benefit from chemotherapy.

## 1 Introduction

Colorectal cancer (CRC) is one of the most common malignant tumors worldwide, seriously threatening human health. According to statistics, its morbidity and mortality rank among the top among various cancers (1). Despite certain progress in the diagnosis and treatment of CRC in recent years, the prognosis of patients with advanced and metastatic CRC is still not ideal, with a low 5-year survival rate (2). Among them, the proficient mismatch repair (pMMR) subtype accounts for approximately 95% of patients with CRC. Because of low tumor mutational burden (TMB) and the lack of immunogenic neoantigens, it usually presents an immunosuppressive microenvironment, with a response rate to immune checkpoint inhibitors (ICIs) of less than 10%, which has become a core bottleneck in clinical treatment (3).

The RAS gene family plays a key role in cell signal transduction pathways, among which KRAS and NRAS gene mutations are common in CRC. The mutation rate of the KRAS gene is approximately 30%–40%, while that of the NRAS gene is relatively low. RAS gene mutations may be related to the poor prognosis of CRC (4, 5). Oncogenic KRAS activates the Wnt signaling pathway in colon cancer by inhibiting GSK-3β, thereby promoting the proliferation, survival, migration, and invasion of tumor cells (6). At the same time, the RAS gene mutation status has an important impact on the selection of treatment strategies and efficacy of CRC. Patients with CRC with KRAS or NRAS gene mutations are usually resistant to anti-EGFR targeted therapies such as cetuximab (7).

In recent years, the research on tumor immune microenvironment (TIME) has attracted much attention. CD4^+^ T cells play roles in antigen presentation, cytokine release, and cytotoxicity (8); CD8^+^ T cells are important cytotoxic T cells that can directly recognize and kill tumor cells (9); CD68^+^ macrophages are often called tumor-associated macrophages (TAMs) in the tumor microenvironment (TME), and CD163 is also one of the markers of TAMs, which is usually considered as a characteristic molecule of M2-type macrophages. TAMs can promote tumor angiogenesis and invasion by secreting cytokines and growth factors, and play a promoting role in tumor growth and metastasis (10). Programmed death-ligand 1 (PD-L1) is an important member of immune checkpoints. As early as 2002, it was found that its high expression on the surface of tumor cells can inhibit the activity of T cells, leading to tumor cells evading the surveillance and killing the body’s immune system (11). CKLF-like MARVEL transmembrane domain containing 6 (CMTM6) has been confirmed as a key regulator of PD-L1, which can stabilize the expression of PD-L1 on the plasma membrane and prevent its degradation by lysosomes, thereby affecting the TIME and immunotherapy effect (12).

In addition, the abnormal activation of the WNT/β-catenin signaling pathway is also an important feature of CRC (13). As the core molecule of this signaling pathway, the abnormal accumulation and nuclear translocation of β-catenin can regulate the expression of a series of target genes and participate in the proliferation, differentiation, migration, and invasion of tumor cells (14). It has also been reported that the WNT/β-catenin signaling pathway plays a key regulatory role in the interaction between cancer cells and TAMs in the TME, and may affect the efficacy of cancer immunotherapy (14). In our previous study, we found that the expression of β-catenin in CRC tissues was positively correlated with the expressions of CMTM6 and PD-L1 (15).

At present, there are few studies on the changes of immune microenvironment in CRC under different K/N-RAS mutation statuses and their correlation with the expressions of β-catenin and CMTM6 proteins. This study aims to comprehensively analyze the relationships between immune microenvironment-related indicators, β-catenin, and CMTM6 protein expressions and clinicopathological features and prognosis of pMMR CRC under different K/N-RAS mutation statuses, and explore the effects of K/N-RAS gene status and immune microenvironment characteristics on chemotherapy efficacy, so as to provide references for the precise diagnosis and treatment of patients with pMMR CRC.

## 2 Materials and methods

### 2.1 Research objects

The clinicopathological data of patients who underwent radical CRC surgery without neoadjuvant chemotherapy in The Affiliated Qingyuan Hospital (Qingyuan People’s Hospital), Guangzhou Medical University from August 2019 to September 2023 were collected, including age, gender, tumor location, tumor type, tumor grade, lymphovascular invasion, perineural invasion, TNM stage, and mismatch repair (MMR) protein expression results. Inclusion criteria were as follows: pathologically confirmed CRC with no loss of MMR protein expression by immunohistochemistry and complete clinical data. Exclusion criteria were as follows: complicated with other malignant tumors; received immunotherapy or chemoradiotherapy before surgery; and had loss of MMR protein expression. This study was approved by the Medical Ethics Committee of The Affiliated Qingyuan Hospital (Qingyuan People’s Hospital), Guangzhou Medical University.

A total of 225 cases were included in the study, including 129 men and 96 women; aged from 29 to 94 years, with a median age of 64 years; 160 cases of left-sided colon (including rectum) and 65 cases of right-sided colon; 176 cases of low-grade (well-differentiated and moderately differentiated adenocarcinoma) tumors and 49 cases of high-grade (poorly differentiated adenocarcinoma and mucinous carcinoma) tumors; 97 cases of TNM stage 1 + 2 and 128 cases of stage 3 + 4. Follow-up was conducted through outpatient clinics, inpatient systems, and telephone calls, and complete follow-up data were collected from 191 cases, with a follow-up rate of 84.89%; 132 patients received postoperative chemotherapy, including 79 cases of XELOX regimen (oxaliplatin + fluoropyrimidines), 35 cases of FOLFOX/FOLFIRI regimen (oxaliplatin/irinotecan + calcium folinate + fluoropyrimidines), 15 cases of fluoropyrimidine monotherapy, and 3 cases of other regimens. Overall survival (OS) was defined as the time from the date of pathological diagnosis of CRC to death from any cause. Patients who were alive at the end of follow-up or lost to follow-up were censored at the date of their last known clinical contact. The data cutoff for survival analysis was 31 December 2024. Vital status was ascertained through institutional electronic medical records.

### 2.2 Experimental methods

The human KRAS/NRAS gene mutation combined detection kit produced by Xiamen AmoyDx Biotech Co., Ltd. was used for PCR detection of K-RAS and N-RAS mutation statuses.

The TMA Master automatic tissue microarray instrument from Beecher Instruments (UK) was used to make microarray arrangements. The fully automatic tissue microarray preparation system was used to drill micropores with a diameter of 2.0 mm in the recipient wax block. For each case, five tissue column samples were collected, including three primary tumor tissues and two adjacent normal mucosal tissues.

Immunohistochemical staining was used to detect the expressions of β-catenin, CMTM6, PD-L1, CD4, CD8, CD68, and CD163 in the tissue microarray. β-catenin rabbit polyclonal antibody, CD4 rabbit polyclonal antibody, CD8 rabbit monoclonal antibody (S118-01), CD68 rabbit polyclonal antibody, and CD163 rabbit polyclonal antibody were purchased from SAB Biotechnology (USA); CMTM6 rabbit monoclonal antibody (EPR23015-45) was purchased from Abcam Biotechnology (USA); PD-L1 immunohistochemical detection reagent (rabbit monoclonal antibody, SP263) was purchased from Roche Diagnostics (Shanghai) Co., Ltd. All immunohistochemical detection experiments were strictly performed in accordance with the reagent instructions on the VENTANA BenchMark Ultra fully automatic immunohistochemical staining instrument; the interpretation of immunohistochemical results was performed by two pathologists separately.

The expressions of β-catenin, CMTM6, and PD-L1 were scored according to staining intensity and positive cell ratio. Staining intensity scoring standard: no staining = 0 point, light yellow = 1 point, tan = 2 points, and brownish black = 3 points, and the number of positive cells was calculated respectively.

The immunohistochemical staining of β-catenin was localized in the cell membrane, cytoplasm, and nucleus, and the percentage of positive cells in different localizations was calculated respectively. Diffuse strong positive in cytoplasm (intensity 3 points) or nuclear staining (intensity ≥1 point) was determined as high expression.

CMTM6 immunohistochemical staining was localized in the cell membrane, intensity ≥1 point was determined as positive, and ≥10% positive cells was determined as high expression.

PD-L1 immunohistochemical staining was localized in the cell membrane; intensity ≥1 point was determined as positive. The percentage of PD-L1-positive tumor cells in all viable tumor cells (PD-L1-TC, ≥3% was determined as high expression) and the percentage of PD-L1-positive tumor-associated immune cells in all tumor-associated immune cells (PD-L1-IC, ≥25% was determined as high expression) were calculated.

CD4 immunohistochemical staining was localized in the cell membrane, intensity ≥1 point was determined as positive, and the percentage of CD4^+^ tumor-associated immune cells in all tumor-associated immune cells was calculated, with ≥1% determined as high infiltration.

CD8 immunohistochemical staining was localized in the cell membrane, CD68 in the cytoplasm, and CD163 in the cell membrane, with intensity ≥1 point determined as positive. The percentage of positive tumor-associated immune cells in all tumor-associated immune cells was calculated, with ≥10% determined as high infiltration; in addition, if CD8^+^ immune cells were observed in the CRC tumor epithelium, it was determined that CD8^+^ T cells had infiltrated the tumor epithelium (CD8-IE).

### 2.3 Data analysis methods

SPSS 25.0 statistical software was used for data analysis. Chi-square test was used to compare the rate differences between groups; Cox regression analysis was used to screen the independent prognostic factors of patients with CRC, including univariate Cox regression analysis and multivariate Cox regression analysis; survival analysis was performed using the Kaplan–Meier method, and the Log-rank test was used to compare the survival differences between different groups. *p* < 0.05 was considered statistically significant.

Pre-specified subgroup analyses were conducted to assess potential heterogeneity in the association between chemotherapy and OS across clinically relevant subgroups. For each subgroup, a likelihood ratio test was used to evaluate the interaction between the subgroup variable and the primary exposure in a Cox proportional hazards (PH) model. Given that only one subgroup interaction reached statistical significance (*p* < 0.05), no adjustment for multiple comparisons was applied.

## 3 Results

### 3.1 K/N-RAS mutation detection results of pMMR CRC

Among 225 patients with pMMR CRC, 119 cases with K/N-RAS mutation were detected, with a mutation rate of 52.89%, and 106 cases of wild type, accounting for 47.11%.

### 3.2 Expressions of immune microenvironment-related markers and β-catenin and CMTM6 proteins in pMMR CRC

**Figure 1:**
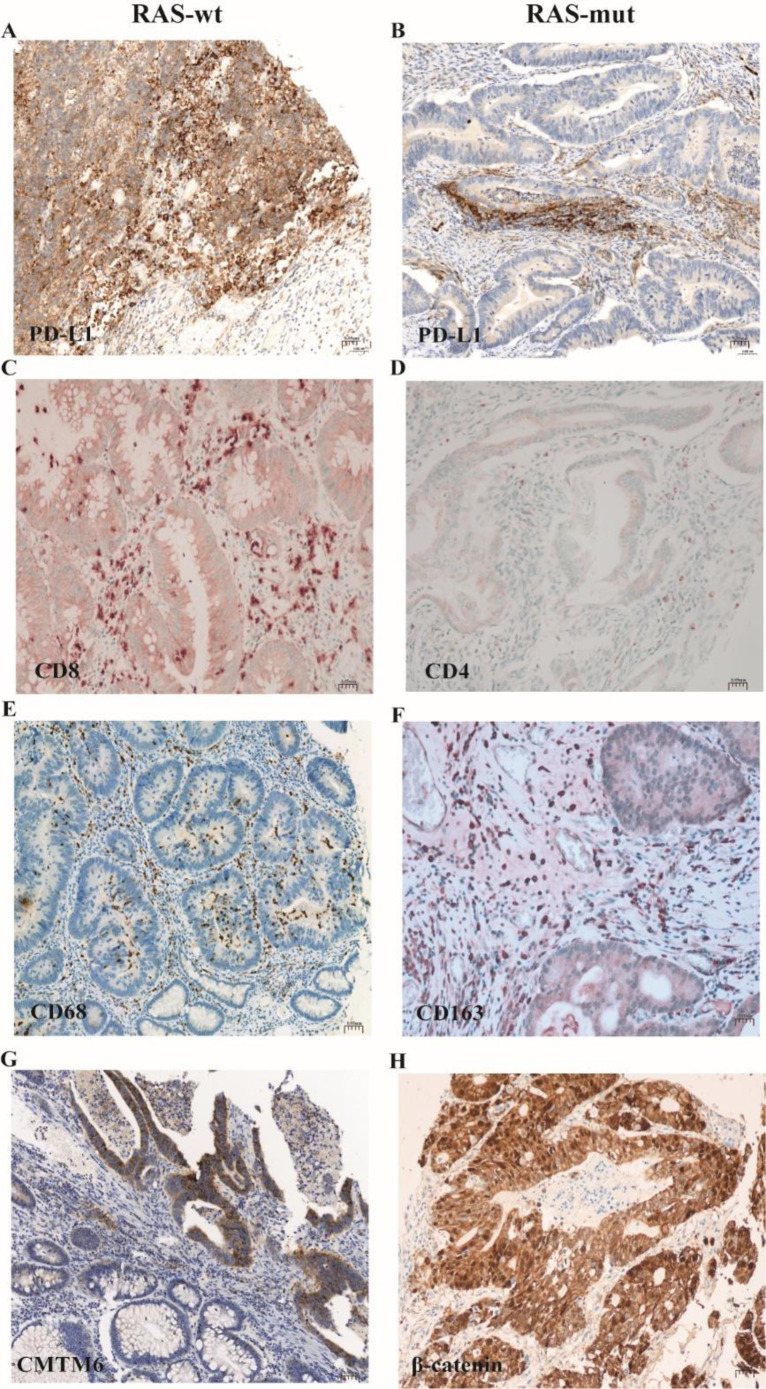
Immunohistochemical expressions of immune microenvironment-related markers and β-catenin and CMTM6 proteins in 225 cases of pMMR CRC. **(A)** PD-L1 expression in CRC tumor epithelium; **(B)** PD-L1 expression in CRC tumor-associated immune cells; **(C)** CD8 expression in CRC tumor stroma; **(D)** CD4 expression in CRC tumor stroma; **(E)** CD68 expression in CRC tumor stroma; **(F)** CD163 expression in CRC tumor stroma; **(G)** CMTM6 expression in CRC; **(H)** β-catenin expression in CRC. (These figures show representative cases.).

As shown in **Figure 1**, PD-L1 immunohistochemical staining was localized in the cell membrane. The high expression rate of PD-L1-TC was 23.1% (52/225, **Figure 1A**); the high expression rate of PD-L1-IC was 18.2% (41/225, **Figure 1B**); CD8 immunohistochemical staining was localized in the cell membrane, the high infiltration rate of CD8^+^ T cells was 52.0% (117/225), and the incidence of CD8^+^ T-cell infiltration in tumor epithelium (CD8-IE) was 48.9% (110/225, **Figure 1C**); CD4 immunohistochemical staining was localized in the cell membrane, and the high infiltration rate of CD4^+^ T cells was 57.8% (130/225, **Figure 1D**); CD68 immunohistochemical staining was localized in the cytoplasm, and the high infiltration rate of CD68^+^ histiocytes was 54.2% (122/225, **Figure 1E**); CD163 immunohistochemical staining was localized in the cell membrane, and the high infiltration rate of CD163^+^ macrophages was 26.2% (59/225, **Figure 1F**); CMTM6 immunohistochemical staining was localized in the cell membrane, and its high expression rate was 34.7% (78/225, **Figure 1G**); β-catenin immunohistochemical staining was localized in the cell membrane, cytoplasm, and nucleus, and its high expression rate was 36.0% (81/225, **Figure 1H**).

### 3.3 Relationship between K/N-RAS mutation status and clinicopathological features and immune microenvironment features

Notably, we observed a significant association between K/N-RAS mutation status and high CD4^+^ T-cell infiltration (*p* = 0.026, **Table 1**); meanwhile, the K/N-RAS mutation rate in patients aged ≥60 years (59.7%) was higher than that in patients aged <60 years (42.9%), with a statistically significant difference (*p* < 0.05); the mutation rate in right-sided colon patients (65.6%) was higher than that in left-sided colon patients (47.5%), with a statistically significant difference (*p* < 0.05); there was no significant correlation between K/N-RAS mutation status and gender, tumor grade, TNM stage, lymphovascular invasion, perineural invasion, as well as the expressions of β-catenin, CMTM6, PD-L1-TC, and PD-L1-IC, and the infiltration degrees of tumor-associated immune cells (CD8^+^ T cells, CD68^+^ histiocytes, and CD163^+^ macrophages) (*p* > 0.05).

**Table 1:** Relationship between K/N-RAS mutation status and clinicopathological features and immune microenvironment in 225 cases of pMMR CRC.

Clinicopathological features	*N* = 225	RAS-wt	RAS-mut	*P*-value
Gender	Female	42 (43.8%)	54 (56.3%)	0.384
	Male	64 (49.6%)	65 (50.4%)	
Age	<60	52 (57.1%)	39 (42.9%)	0.013
	≥60	54 (40.3%)	80 (59.7%)	
Tumor location	Right	22 (33.8%)	43 (66.2%)	0.011
	Left	84 (52.5%)	76 (47.5%)	
Grade	Low	85 (48.3%)	91 (51.7%)	0.501
	High	21 (42.9%)	28 (57.1%)	
TNM_stage	1 + 2	42 (43.3%)	55 (56.7%)	0.319
	3 + 4	64 (50.0%)	64 (50.0%)	
Lymphovascular Invasion	No	73 (44.2%)	92 (55.8%)	0.123
	Yes	33 (55.9%)	26 (44.1%)	
Perineural invasion	No	53 (46.9%)	60 (53.1%)	0.899
	Yes	53 (47.7%)	58 (52.3%)	
β-catenin	Low	68 (47.2%)	76 (52.8%)	0.964
	High	38 (46.9%)	43 (53.1%)	
CMTM6	Low	72 (49.0%)	75 (51.0%)	0.441
	High	34 (43.6%)	44 (56.4%)	
PD-L1-TC	Low	87 (50.3%)	86 (49.7%)	0.082
	High	19 (36.5%)	33 (63.5%)	
PD-L1-IC	Low	84 (45.7%)	100 (54.3%)	0.353
	High	22 (53.7%)	19 (46.3%)	
CD4^+^	Low	53 (55.8%)	42 (44.2%)	0.026
	High	53 (40.8%)	77 (59.2%)	
CD8^+^	Low	47 (43.5%)	61 (56.5%)	0.300
	High	59 (50.4%)	58 (49.6%)	
CD8-IE	No	55 (47.8%)	60 (52.2%)	0.826
	Yes	51 (46.4%)	59 (53.6%)	
CD68^+^	Low	44 (42.7%)	59 (57.3%)	0.225
	High	62 (50.8%)	60 (49.2%)	
CD163^+^	Low	81 (48.8%)	85 (51.2%)	0.396
	High	25 (42.4%)	34 (57.6%)	

RAS-wt, K/N-RAS wild type; RAS-mut, K/N-RAS mutant type.

We analyzed a microsatellite stable (MSS) CRC cohort from The Cancer Genome Atlas (TCGA), including 372 cases with available RAS status (176 RAS mutant and 196 RAS wild type). Gene Set Enrichment Analysis (GSEA) was performed to evaluate the association between RAS mutation and CD8^+^ T-cell function-related gene signatures. Notably, both the “CD8^+^ T cell vs. B2 B-cell upregulated gene set” and the “ALPHAALPHA vs. ALPHABETA CD8^+^ T cell downregulated gene set” were significantly negatively enriched in RAS-mutant samples compared with RAS wild-type samples (Enrichment Score < 0, **Supplementary Figure 1**), with peak enrichment occurring in the latter half of the ranked gene list (corresponding to regions of high expression in RAS wild-type samples). These findings indicate that RAS mutation is associated with global suppression of CD8^+^ T-cell effector gene signatures in MSS CRC, which may contribute to the formation of an immunosuppressive TME and impair anti-tumor immune responses.

### 3.4 Prognostic analysis of patients with pMMR CRC with different K/N-RAS statuses

Among 191 patients with pMMR CRC with complete follow-up data, the median follow-up time was 44 months (2–66 months), the 3-year OS rate was 81.0%, and the 5-year OS rate was 72.4%. The 3-year OS rate of K/N-RAS wild-type patients was 82.3%, and the 5-year OS rate was 73.6%; the 3-year OS rate of K/N-RAS mutant patients was 79.8%, and the 5-year OS rate was 71.5%. There was no significant difference in OS between the two groups (*p* > 0.05).

We verified the PH assumption for all covariates in the two Cox models (K/N-RAS wild-type group and K/N-RAS mutant group) via Schoenfeld residuals using the cox.zph() function (survival package, R 4.5.2). Although the test for lymphovascular invasion yielded a statistically significant result (*p* < 0.05) in the K/N-RAS wild-type model, its residual plot showed no distinct time-dependent trend, so it was included as a time-constant effect. All variables in the K/N-RAS mutant model satisfied the PH assumption (all *p* > 0.05).

Cox regression analysis was performed on 89 patients with pMMR and K/N-RAS wild-type CRC. As shown in **Table 2**, univariate analysis results showed that patients with pMMR CRC aged ≥60 years, with right-sided colon, low CD8^+^ T-cell infiltration, and low CD68^+^ histiocyte infiltration had poor prognosis (*p* < 0.05); multivariate analysis results showed that age ≥60 years, right-sided colon, TNM stage 3 + 4, low CD4^+^ T-cell infiltration, and low CD68^+^ histiocyte infiltration were independent prognostic factors for poor prognosis (*p* < 0.05). (In the multivariate analysis, the variable CD8^+^ T-cell infiltration lost its statistical significance, which may be attributed to multicollinearity with other stronger prognostic factors. After adjusting for confounding factors, its independent prognostic value disappeared).

**Table 2:** COX regression analysis of 89 patients with pMMR and K/N-RAS wild-type CRC.

Clinicopathological factors	OS (Univariate analysis)			OS (Multivariate analysis)		
	95% CI	HR	*p*	95% CI	HR	*p*
Gender (male vs. female)	0.309–1.876	0.762	0.554	0.224–3.131	0.837	0.792
Age (≥60 vs. <60)	1.081–8.337	3.002	0.035	1.952–19.083	6.103	0.002
Tumor location (left vs. right)	0.122–0.852	0.322	0.022	0.079–0.620	0.221	0.004
Grade (high vs. low)	0.274–3.243	0.943	0.926	0.050–1.622	0.283	0.157
Lymphovascular Invasion (yes vs. no)	0.635–4.120	1.618	0.313	0.399–7.905	1.775	0.451
Perineural invasion (yes vs. no)	0.652–4.214	1.658	0.288	0.485–5.705	1.663	0.418
TNM stage (3 + 4 stage vs. 1 + 2 stage)	0.956–8.717	2.887	0.060	2.607–38.790	10.057	0.001
β-catenin (high vs. low)	0.251–1.935	0.697	0.488	0.196–3.202	0.792	0.744
CMTM6 (high vs. low)	0.469–3.246	1.234	0.671	0.359–4.855	1.32	0.676
PD-L1-TC (high vs. low)	0.394–3.577	1.187	0.761	0.381–13.122	2.236	0.373
PD-L1-IC (high vs. low)	0.595–4.593	1.653	0.335	0.096–1.326	2.092	0.389
CD4^+^ (high vs. low)	0.279–1.722	0.693	0.429	0.119–0.978	0.341	0.045
CD8^+^ (high vs. low)	0.144–0.998	0.379	0.049	0.158–1.863	0.543	0.332
CD8-IE (yes vs. no)	0.359–2.223	0.894	0.809	0.154–3.044	0.684	0.618
CD68^+^ (high vs. low)	0.152–0.979	0.386	0.045	0.086–0.624	0.231	0.004
CD163^+^ (high vs. low)	0.544–3.771	1.433	0.467	0.345–3.266	1.062	0.917

Cox regression analysis was performed on 102 patients with pMMR and K/N-RAS mutant CRC. As shown in **Table 3**, univariate analysis results showed that patients aged ≥60 years and those with high CD163^+^ macrophage infiltration had poor prognosis (*p* < 0.05); multivariate analysis results showed that age ≥60 years, TNM stage 3 + 4, absence of CD8^+^ T-cell infiltration in tumor epithelium, and high CD163^+^ macrophage infiltration were independent prognostic factors for poor prognosis (*p* < 0.05).

**Table 3:** COX regression analysis of 102 patients with pMMR and K/N-RAS mutant CRC.

Clinicopathological factors	OS (Univariate analysis)			OS (Multivariate analysis)		
	95% CI	HR	*p*	95% CI	HR	*p*
Gender (male vs. female)	0.626–3.206	1.417	0.403	0.402–2.556	1.013	0.978
Age (≥60 vs. <60)	1.438–16.063	4.806	0.011	1.272–14.492	4.294	0.019
Tumor location (left vs. right)	0.504–2.712	1.170	0.715	0.321–2.895	0.965	0.949
Grade (high vs. low)	0.977–5.271	2.270	0.057	0.469–3.702	1.318	0.601
Lymphovascular Invasion (yes vs. no)	0.785–4.512	1.882	0.156	0.498–5.343	1.631	0.419
Perineural invasion (yes vs. no)	0.862–4.419	1.952	0.109	0.356–2.218	0.889	0.800
TNM stage (stage 3 + 4 vs. stage 1 + 2)	0.995–5.357	2.309	0.051	1.169–6.596	2.777	0.021
β-catenin (high vs. low)	0.652–3.168	1.437	0.368	0.664–4.073	1.645	0.282
CMTM6 (high vs. low)	0.477–2.375	1.065	0.878	0.283–2.151	0.781	0.632
PD-L1-TC (high vs. low)	0.691–3.544	1.565	0.283	0.662–5.000	1.820	0.245
PD-L1-IC (high vs. low)	0.026–1.428	0.193	0.107	0.027–1.618	0.209	0.134
CD4^+^ (high vs. low)	0.463–2.375	1.049	0.909	0.492–3.221	1.258	0.632
CD8^+^ (high vs. low)	0.416–2.000	0.912	0.819	0.636–4.160	1.627	0.310
CD8-IE (yes vs. no)	0.191–1.025	0.442	0.057	0.149–0.896	0.366	0.028
CD68^+^ (high vs. low)	0.277–1.374	0.617	0.237	0.175–1.197	0.458	0.111
CD163^+^ (high vs. low)	1.439–6.931	3.158	0.004	1.574–8.327	3.621	0.002

### 3.5 Relationships between immune microenvironment-related markers and β-catenin and CMTM6 protein expressions under different K/N-RAS statuses

As shown in **Table 4**, in the K/N-RAS wild-type state, there was no significant correlation between β-catenin and immune microenvironment-related markers (*p* > 0.05), while the high expression rate of PD-L1-TC and the high infiltration rate of CD68^+^ histiocytes in the CMTM6 high-expression group were significantly higher than those in the CMTM6 low-expression group (*p* < 0.05).

**Table 4:** Relationships between immune microenvironment-related markers and β-catenin and CMTM6 protein expressions in K/N-RAS wild-type pMMR CRC (*n* = 106).

TIME	*n* = 106	β-catenin			CMTM6		
		Low	High	*P*-value	Low	High	*P*-value
PD-L1-TC	Low	58 (66.7%)	29 (33.3%)	0.248	64 (73.6%)	23 (26.4%)	0.008
	High	10 (52.6%)	9 (47.4%)		8 (42.1%)	11 (57.9%)	
PD-L1-IC	Low	53 (63.1%)	31 (36.9%)	0.658	56 (66.7%)	28 (33.3%)	0.588
	High	15 (68.2%)	7 (31.8%)		16 (72.7%)	6 (27.3%)	
CD4^+^	Low	37 (69.8%)	16 (30.2%)	0.224	34 (64.2%)	19 (35.8%)	0.405
	High	31 (58.5%)	22 (41.5%)		38 (71.7%)	15 (28.3%)	
CD8^+^	Low	34 (72.3%)	13 (27.7%)	0.117	33 (70.2%)	14 (29.8%)	0.652
	High	34 (57.6%)	25 (42.4%)		39 (66.1%)	20 (33.9%)	
CD8-IE	No	32 (58.2%)	23 (41.8%)	0.183	37 (67.3%)	18 (32.7%)	0.881
	Yes	36 (70.6%)	15 (29.4%)		35 (68.6%)	16 (31.4%)	
CD68^+^	Low	30 (68.2%)	14 (31.8%)	0.466	35 (79.5%)	9 (20.5%)	0.031
	High	38 (61.3%)	24 (38.7%)		37 (59.7%)	25 (40.3%)	
CD163^+^	Low	51 (63.0%)	30 (37.0%)	0.646	55 (67.9%)	26 (32.1%)	0.993
	High	17 (68.0%)	8 (32.0%)		17 (68.0%)	8 (32.0%)	
β-catenin	Low				50 (73.5%)	18 (26.5%)	0.098
	High				22 (57.9%	16 (42.1%)	
CMTM6	Low	50 (69.4%)	22 (30.6%)	0.098			
	High	18(52.9%)	16(47.1%)				

As shown in **Table 5**, in the K/N-RAS mutant state, the high expression rate of CMTM6 and the high infiltration rate of CD163^+^ macrophages in the β-catenin high-expression group were significantly higher than those in the β-catenin low-expression group (*p* < 0.05); the high expression rate of PD-L1-TC in the CMTM6 high-expression group was significantly higher than that in the CMTM6 low-expression group (*p* < 0.05).

**Table 5:** Relationships between immune microenvironment-related markers and β-catenin and CMTM6 protein expressions in K/N-RAS mutant pMMR CRC (*n* = 119).

TIME	*n* = 119	β-catenin			CMTM6		
		Low	High	*P*-value	Low	High	*P*-value
PD-L1-TC	Low	59 (68.6%)	27 (31.4%)	0.082	59 (68.6%)	27 (31.4%)	0.042
	High	17 (51.5%)	16 (48.5%)		16 (48.5%)	17 (51.5%)	
PD-L1-IC	Low	65 (65.0%)	35 (35.0%)	0.555	64 (64.0%)	36 (36.0%)	0.613
	High	11 (57.9%)	8 (42.1%)		11 (57.9%)	8 (42.1%)	
CD4^+^	Low	27 (64.3%)	15 (35.7%)	0.944	28 (66.7%)	14 (33.3%)	0.543
	High	49 (63.6%)	28 (36.4%)		47 (61.0%)	30 (39.0%)	
CD8^+^	Low	42 (68.9%)	19 (31.1%)	0.246	40 (65.6%)	21 (34.4%)	0.555
	High	34 (58.6%)	24 (41.4%)		35 (60.3%)	23 (39.7%)	
CD8-IE	No	38 (63.3%)	22 (36.7%)	0.903	37 (61.7%)	23 (38.3%)	0.757
	Yes	38 (64.4%)	21 (35.6%)		38 (64.4%)	21 (35.6%)	
CD68^+^	Low	38 (64.4%)	21 (35.6%)	0.903	41 (69.5%)	18 (30.5%)	0.147
	High	38 (63.3%)	22 (36.7%)		34 (56.7%)	26 (43.3%)	
CD163^+^	Low	59 (69.4%)	26 (30.6%)	0.046	55 (64.7%)	30 (35.3%)	0.548
	High	17 (50.0%)	17 (50.0%)		20 (58.8%)	14 (41.2%)	
β-catenin	Low				53 (69.7%)	23 (30.3%)	0.044
	High				22 (51.2%)	21 (48.8%)	
CMTM6	Low	53 (70.7%)	22 (29.3%)	0.044			
	High	23(52.3%)	21(47.1%)				

### 3.6 Effects of K/N-RAS status and immune features on chemotherapy benefit in stage 3 + 4 pMMR CRC

While RAS mutation status did not serve as a significant prognostic factor for OS in the entire cohort, we hypothesized that it might act as a predictive biomarker for chemotherapy response.

**Figure 2:**
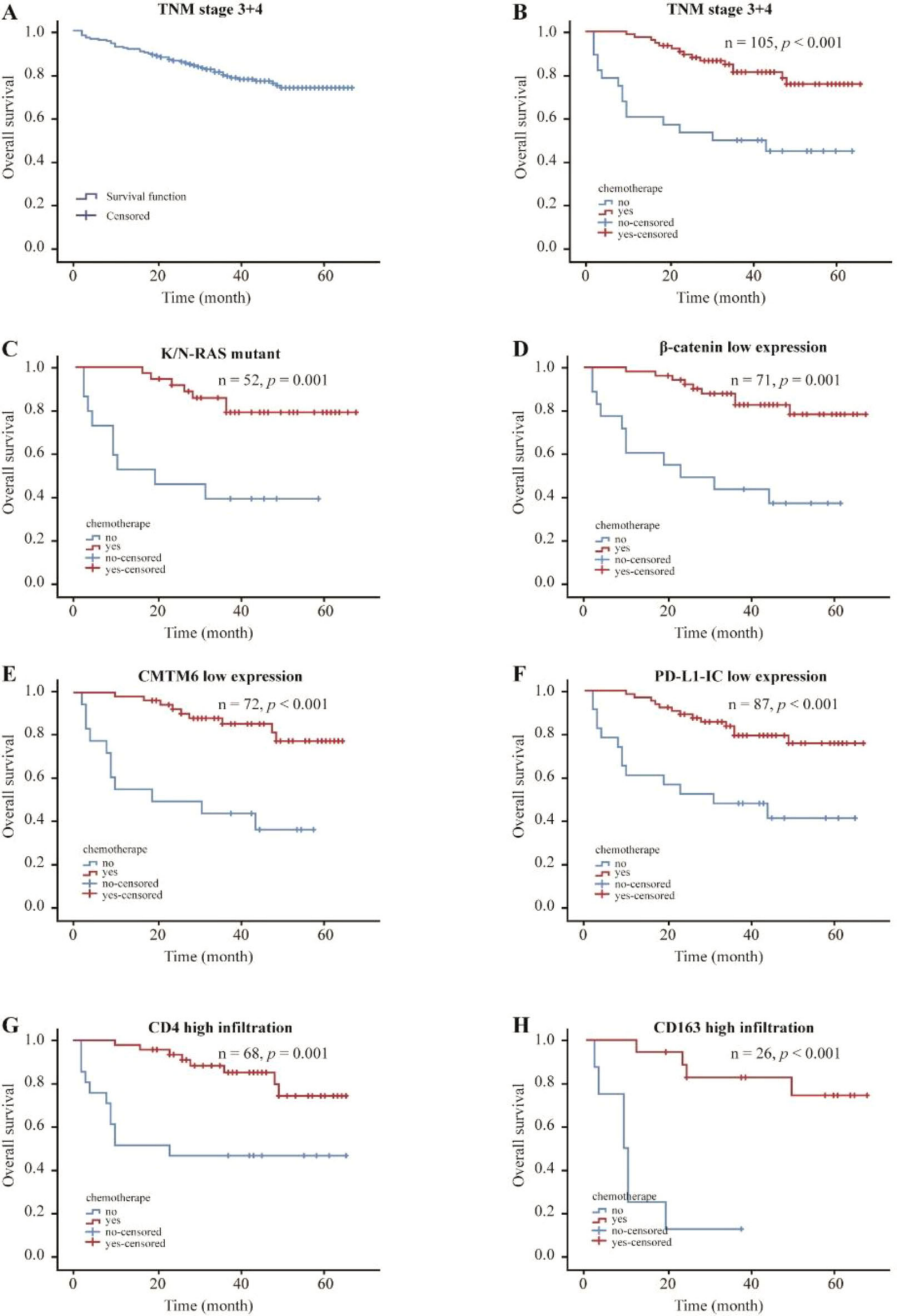
Analysis of the effect of chemotherapy on the survival time of patients with TNM stage 3 + 4 pMMR CRC. **(A)** Overall survival curve of 105 cases; **(B)** 105 cases of TNM stage 3 + 4; **(C)** 52 cases with K/N-RAS mutant; **(D)** 71 cases with β-catenin low expression; **(E)** 72 cases with CMTM6 low expression; **(F)** 87 cases with PD-L1-IC low expression; **(G)** 68 cases with CD4^+^ T-cell high infiltration; **(H)** 26 cases with CD163^+^ macrophage high infiltration.

This study analyzed the effect of chemotherapy on the survival time of 105 patients with pMMR CRC with TNM stage 3 + 4 and complete follow-up data. As shown in **Figure 2B**, the survival time of the chemotherapy group was significantly longer than that of the non-chemotherapy group in patients with TNM stage 3 + 4 pMMR CRC (*p* < 0.001). Stratified analysis results demonstrate that the survival benefit of chemotherapy was not uniform, but was instead stratified by the molecular-immune landscape defined by RAS status and specific immune markers. The survival time of the chemotherapy group was significantly longer than that of the non-chemotherapy group in the K/N-RAS mutant group, β-catenin low-expression group, CMTM6 low-expression group, PD-L1-IC low-expression group, CD4^+^ T-cell high-infiltration group, and CD163^+^ macrophage high-infiltration group (**Figure 2**, *p* < 0.05), but there was no significant difference in survival time between the chemotherapy group and the non-chemotherapy group in the K/N-RAS wild-type group, β-catenin high-expression group, CMTM6 high-expression group, PD-L1-IC high-expression group, CD4^+^ T-cell low-infiltration group, and CD163^+^ macrophage low-infiltration group (**Supplementary Figure 2**) (*p* > 0.05).

**Figure 3:**
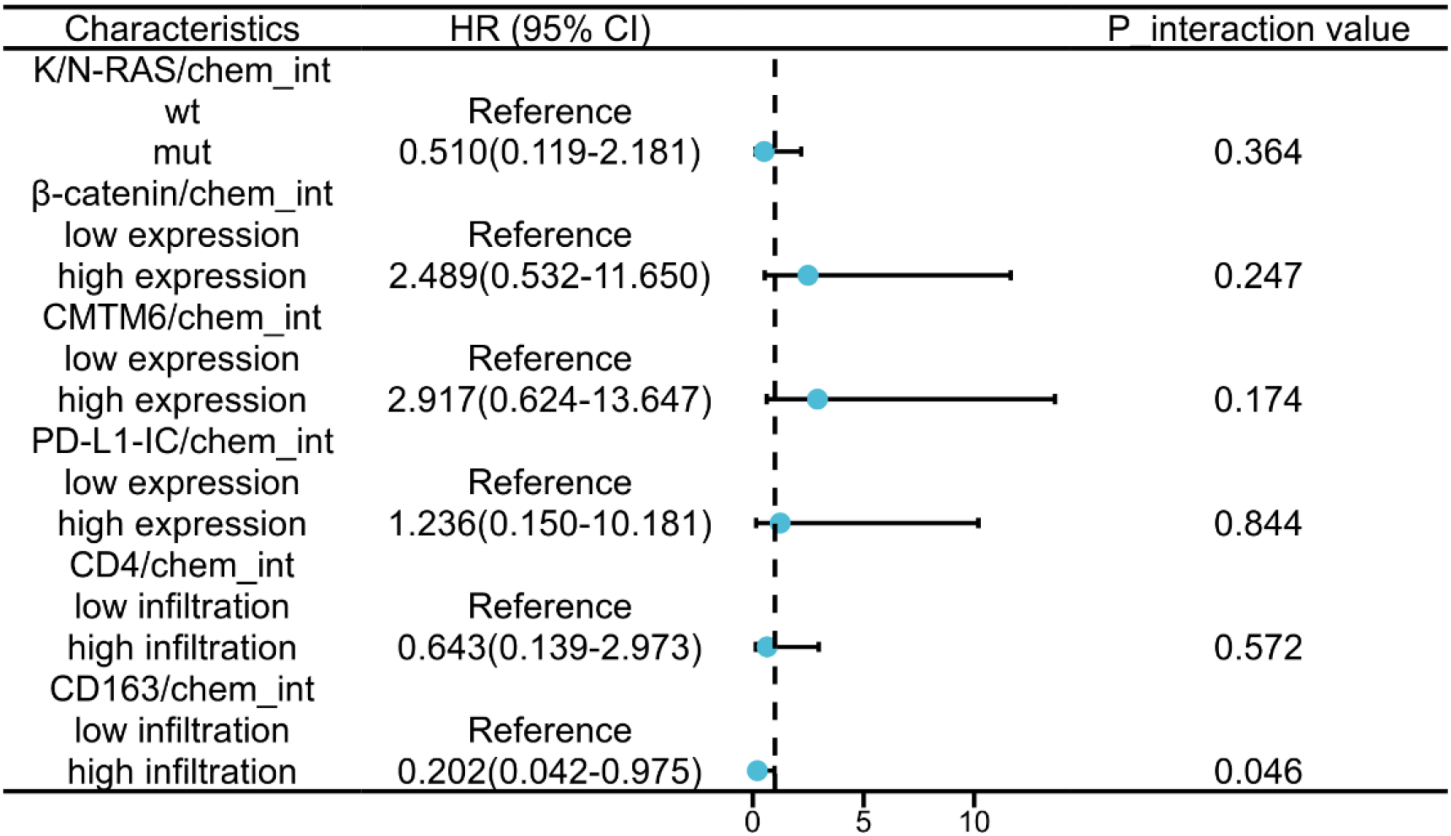
Forest plot of subgroup analyses for the effect of chemotherapy on overall survival. Forest plot demonstrated heterogeneous chemotherapy effects across subgroups, with significant interaction only for CD163 infiltration status (*p* interaction = 0.046).

To explore whether the survival benefit of chemotherapy varied across different molecular and immune subgroups, we performed stratified Cox PH analyses. We evaluated the treatment effect within subgroups defined by K/N-RAS status; β-catenin, CMTM6, and PD-L1-IC expression levels; and CD163 and CD4 infiltration levels. Crucially, we included an interaction term (Chemotherapy × Biomarker) in the models to formally test for heterogeneity of the treatment effect between subgroups. A statistically significant interaction was observed for CD163^+^ infiltration level, indicating that the efficacy of chemotherapy differed substantially based on CD163^+^ macrophage infiltration levels. In patients with CD163^+^ macrophage high infiltration, chemotherapy was associated with a marked improvement in OS compared to the control group (HR = 0.202, 95% CI: 0.04–0.98, *p* interaction = 0.046, **Figure 3**). In contrast, no significant survival benefit was observed in patients with low CD163^+^ macrophage infiltration. This result suggests that CD163^+^ macrophage high infiltration serves as a predictive biomarker for favorable response to chemotherapy in pMMR CRC.

In both the low CD8^+^ T-cell infiltration group and the high CD8^+^ T-cell infiltration group, with CD8^+^ T-cell infiltration into the tumor epithelium group and without CD8^+^ T-cell infiltration into the tumor epithelium group, survival time was significantly longer in patients who received chemotherapy than in those without chemotherapy (**Supplementary Figures 3A–D**)(*p* < 0.05).

We performed a stratified survival analysis of chemotherapy according to K/N-RAS status in patients with high CD4^+^ T-cell infiltration. The results showed that in the high CD4^+^ T-cell infiltration subgroup, chemotherapy significantly improved survival in both K/N-RAS wild-type and mutant patients (**Supplementary Figures 3E, F**) (*p* < 0.05).

## 4 Discussion

The analysis of the pMMR CRC population in this study showed that the mutation rate in patients aged ≥60 years was significantly higher than that in the <60-year group, and the mutation rate in right-sided colon tumors was significantly higher than that in left-sided colon tumors. Kundranda et al. (16) also found that RAS mutations mostly occurred in patients >50 years old. A systematic review and meta-analysis by Bylsma et al. (17) covering 44 studies with a total of 15,981 patients with metastatic CRC also found that the mutation rates of NRAS and KRAS in right-sided tumors were significantly higher than those in left-sided tumors. The potential reason for this difference may be related to the different embryonic origins of the left and right colon: the right colon originates from the midgut, while the left colon and rectum originate from the hindgut. Cells of different embryonic origins may have differences in proliferation patterns, differentiation pathways, and signal transduction pathways, which may provide an internal molecular basis for the difference in K/N-RAS mutation rates (18). This study also found that the infiltration degree of CD4^+^ T cells in the tumor stroma of the RAS mutant group was higher, which suggests that RAS mutation may selectively affect the infiltration pattern of specific immune cell subsets.

Cox regression analysis showed that regardless of whether K/N-RAS was in mutant or wild-type state, age ≥60 years and TNM stage 3 + 4 were independent poor prognostic factors for patients with pMMR CRC. The clinical value of TNM stage as a classic prognostic indicator for CRC has been fully verified, while the reduced survival time of elderly patients may be affected by other factors such as combined underlying diseases and physical function decline (19). In RAS wild-type cases, right-sided colon tumors, low CD4^+^ T-cell infiltration, and low CD68^+^ histiocyte infiltration were independent poor prognostic factors. Studies (20) have also found that in the RAS wild-type population, the progression-free survival (PFS), OS, and objective response rate (ORR) of patients with right-sided colon tumors were significantly worse than those of patients with left-sided colon tumors. Studies (21) have also found that patients with CRC with low CD4^+^ T-cell infiltration have poor prognosis. A study by Jiang et al. indicated that the infiltration of Foxp3^+^ regulatory T cells (Tregs) was increased in patients with Ras-mutated dMMR CRC (22). We speculate that the increased CD4^+^ T cells observed in the present study may be enriched in the Treg subset, which explains their association with poor prognosis. A study (23) on 196 patients with CRC found that the low infiltration density of CD68^+^ cells was an independent predictor of poor prognosis. The protective effect of CD4^+^ T cells is related to their immune regulation function, while in CD68^+^ histiocytes, as important phagocytes and antigen-presenting cells, insufficient infiltration may lead to antigen presentation defects, thereby weakening the body’s anti-tumor immune response (24). In the RAS mutant population, the absence of CD8^+^ T-cell infiltration in tumor epithelium and high CD163^+^ macrophage infiltration were independent poor prognostic factors. As cytotoxic effector cells, the infiltration of CD8^+^ T cells in tumor epithelium indicates a good prognosis. CD163^+^ macrophages are mostly M2-type macrophages with immunosuppressive functions, and their high infiltration will further aggravate the immunosuppressive state of the TME, which is consistent with the characteristic of RAS mutant tumors being prone to immune escape reported in previous studies (25). The above differences reveal that RAS mutation can affect the TIME of CRC: the progression of wild-type tumors depends more on the intensity of the immune response, while mutant tumors achieve immune escape by remodeling the immune microenvironment (22, 26). The results of this study can provide clinical data reference for the subsequent formulation of individualized immunotherapy strategies for patients with pMMR CRC with different RAS statuses.

As the core effector molecule of the Wnt pathway, the abnormal activation of β-catenin is a key event in the occurrence and development of CRC (13). β-catenin can regulate its stability by directly binding to specific domains of RAS protein, while RAS mutation will destroy this interaction and the subsequent degradation process, leading to the synergistic stability of β-catenin and mutant RAS protein (27). RAS mutation can inhibit the activity of GSK-3β through the PI3K/AKT pathway, thereby stabilizing β-catenin protein and promoting its nuclear translocation (7). RAS mutation can also regulate the M2-type polarization of macrophages by secreting cytokines such as CSF2 and interleukin-6 (IL-6) (28). This study found that regardless of RAS wild-type or mutant status, the high expression rate of PD-L1 on tumor cells (PD-L1-TC) was significantly higher in cases with high CMTM6 expression than in those with low CMTM6 expression, which is consistent with our previous findings (15). Notably, in the RAS-mutant pMMR CRC subgroup, the proportion of cases with high CMTM6 expression was markedly increased in patients with elevated β-catenin expression, accompanied by high infiltration of CD163^+^ macrophages; this phenomenon was not observed in the RAS wild-type subgroup. Our previous tissue microarray analysis of 704 patients with CRC demonstrated that CMTM6 positive expression was significantly positively correlated with β-catenin expression, especially prominent in the pMMR subgroup. Relevant studies on head and neck squamous cell carcinoma (29) have also validated the positive correlation between CMTM6 expression and the activity of the Wnt/β-catenin signaling pathway. Pang et al. indicated in studies of oral squamous cell carcinoma that exosomes carrying CMTM6 secreted by tumor cells can be internalized by macrophages, thereby inducing M2 macrophage polarization via activation of the ERK1/2 signaling pathway (30). Other studies have shown that PD-L1 expression is significantly associated with the infiltration of CD163^+^ TAMs in cervical cancer, and M2-type TAMs can upregulate PD-L1 expression in cervical cancer cells through the PI3K/AKT signaling pathway (31). In addition, it has been reported that the combination of PD-L1 blockade therapy and CAR-T cell therapy can promote macrophage polarization toward the M1 phenotype and deplete CD163^+^ M2 macrophages via the interferon-γ signaling axis, thereby enhancing the anti-tumor activity of CAR-T cells (32). Another study on cutaneous T-cell lymphoma revealed that combined anti-PD-L1 therapy and lenalidomide could reprogram PD-1-positive M2-like macrophages, reduce CD163 expression, and amplify immune responses (33). Based on the above results and supporting literature, we hypothesized that in RAS-mutant pMMR CRC, activated RAS signaling inhibits GSK-3β activity through the PI3K/AKT/GSK-3β axis, stabilizes β-catenin, and promotes its nuclear translocation. Intranuclear β-catenin may upregulate CMTM6 expression by enhancing the transcriptional activity of the Wnt pathway. Highly expressed CMTM6 shapes an immunosuppressive TME through dual mechanisms. On the one hand, inside tumor cells, CMTM6 binds to PD-L1, inhibits its lysosomal degradation, maintains high PD-L1 expression on the tumor cell surface, and directly suppresses the function of CD8^+^ T cells. On the other hand, referring to the findings of Pang et al. in oral squamous cell carcinoma, CMTM6-positive exosomes secreted by tumor cells are engulfed by macrophages and activate the ERK1/2 pathway to trigger M2-type (CD163^+^) polarization. The significant correlation between high CMTM6 expression and CD163^+^ macrophage infiltration observed in this study provides indirect evidence for this inference. Polarized M2 macrophages further jointly inhibit T-cell immune responses by secreting IL-10 and transforming growth factor-β (TGF-β) and expressing PD-L1 (34). A study on melanoma has confirmed a significant positive correlation between CMTM6 protein expression and CD68^+^ histiocyte infiltration (35). Consistently, our results showed high infiltration of CD68^+^ histiocytes in patients with pMMR CRC with high CMTM6 expression under the RAS wild-type background. These findings suggest that in the absence of potent oncogenic driver mutations (such as RAS mutations), CMTM6 may recruit pan-macrophage populations by regulating the stress status of tumor cells or the antigen-presenting microenvironment. In conclusion, the dual mechanisms of CMTM6-mediated PD-L1 stabilization and M2 macrophage polarization may collectively constitute a key regulatory node of the immunosuppressive microenvironment in RAS-mutant pMMR CRC, and the detailed molecular mechanisms require further experimental verification. Meanwhile, the CMTM6-driven immunosuppressive microenvironment may synergistically promote chemotherapy resistance through dual pathways of immune escape and direct pro-survival effects. Therefore, targeting CMTM6, combined with immune microenvironment modulation (e.g., PD-L1 inhibitors) and standard chemotherapy, may reverse immunosuppression and enhance chemosensitivity. This provides a potential combination therapeutic strategy for pMMR patients who exhibit poor responses to single-agent immunotherapy. Nevertheless, the impact of RAS status must be considered when evaluating CMTM6 as a therapeutic target.

Our study demonstrates that chemotherapy significantly improves OS in patients with stage 3 + 4 pMMR CRC, but this benefit is strictly confined to specific molecular-immune subgroups. Notably, formal interaction testing confirmed that high stromal infiltration of CD163^+^ macrophages is a robust predictive biomarker: patients with high CD163^+^ infiltration levels derived substantial survival benefit from chemotherapy (HR = 0.202), whereas those with low infiltration did not. This finding appears paradoxical given that CD163^+^ macrophages are typically associated with an immunosuppressive, M2-like phenotype. However, this context-dependent role can be reconciled by the distinct effects of platinum-based regimens—the backbone of CRC treatment in our cohort—on the TIME. Preclinical evidence indicates that oxaliplatin can repolarize TAMs toward an immunostimulatory, M1-like state (36, 37), suggesting that a pre-existing high density of CD163^+^ macrophages may serve as a responsive substrate for such therapeutic reprogramming. In contrast, in triple-negative breast cancer treated with anthracycline/taxane-based regimens—which can promote M2 polarization via NF-κB activation (38)—high CD163^+^ infiltration predicts poor chemotherapy response (38). Thus, the predictive value of TIME features is intrinsically linked to both tumor type and the specific chemotherapeutic agents employed.

Beyond CD163, survival benefit was also enriched in patients with K/N-RAS mutations, low β-catenin or CMTM6 expression, low PD-L1 on immune cells (PD-L1-IC), and high CD4^+^ T-cell infiltration. The favorable impact of CD4^+^ T cells was observed irrespective of RAS status, underscoring their central role in coordinating anti-tumor immunity and potentially synergizing with chemotherapy-induced immunogenic cell death (39). Conversely, aberrant activation of the Wnt/β-catenin pathway—often downstream of mutant RAS—and its regulator CMTM6 has been implicated in mediating chemoradioresistance through cancer stem cell maintenance and immunosuppression (40–42). Similarly, high PD-L1-IC may reflect an adaptive immune resistance mechanism that blunts the efficacy of cytotoxic therapy alone (43).

Collectively, these data argue that chemotherapy sensitivity in pMMR CRC is not uniform but is shaped by an integrated molecular-immune signature. Integrating RAS status with key components of the TIME may enable more precise patient selection for chemotherapy, thereby avoiding ineffective treatment in non-responders.

Our findings suggest a pragmatic clinical algorithm: for patients with stage III pMMR CRC, assessment of RAS status and CD163^+^ macrophage infiltration could stratify patients into distinct risk/benefit categories. RAS-mutant/CD163-high patients derive exceptional benefit from adjuvant chemotherapy, justifying aggressive treatment even in borderline-fit patients. Conversely, RAS wild-type/CD163-low patients may be candidates for chemotherapy de-escalation trials.

## 5 Conclusions

In conclusion, K/N-RAS status defines two distinct subtypes of pMMR CRC with divergent immune microenvironments and chemotherapy responses. RAS wild-type tumors benefit from high CD4^+^ T-cell infiltration, whereas RAS-mutant tumors—despite a more immunosuppressive profile characterized by CD8^+^ T-cell exclusion and CD163^+^ macrophage abundance—derive significant survival benefit from chemotherapy. Notably, high CD163^+^ infiltration was validated as a predictive biomarker for chemotherapy response. These findings highlight that the prognostic and predictive impact of the TIME is shaped by oncogenic context, supporting an integrated RAS-immune stratification to guide personalized therapy in pMMR CRC.

## 6 Limitations of this study

This study is a single-center retrospective analysis, which may be subject to selection bias and unmeasured confounding factors. Because of the limited sample size, we were unable to stratify patients by specific K/N-RAS mutation subtypes (e.g., G12D and G13D), despite evidence suggesting that different RAS variants may exhibit distinct biological and immunomodulatory properties. Furthermore, CD4^+^ T-cell subsets—particularly Foxp3^+^ regulatory T cells—were not comprehensively characterized, limiting our ability to fully dissect the complexity of the immune microenvironment. These aspects will be addressed in future large-scale, multicenter studies.

## Data Availability

The raw data supporting the conclusions of this article will be made available by the authors, without undue reservation.
