# Supplementary material for: RAS mutation status and immune microenvironment define distinct prognostic landscapes and predict chemotherapy benefit in pMMR colorectal cancer
Source: Front Immunol. 2026 May 7;17:1798858. doi: 10.3389/fimmu.2026.1798858 (PMC13189948; doi:10.3389/fimmu.2026.1798858)
Supplement: Supplementary file 1 [file Table1.docx]

> library(survival)

> library(survminer)

> mydata <- read.csv("RAS_mu_CRC102.csv") or（"RAS_wi_CRC89.csv "）

> mydata <- na.omit(mydata)

> cox_model <- coxph(

+ Surv(time, status) ~ gender + age + locate + Grade + lymphovasicular + Perineural + TNMstage + βcatenin + CMTM6 + PDL1TC + PDL1IC + CD4 + CD8 + CD8IE + CD68 + CD163,

+ data = mydata

+ )

> test_ph <- cox.zph(cox_model)

> print(test_ph)
