# Supplementary material for: RAS mutation status and immune microenvironment define distinct prognostic landscapes and predict chemotherapy benefit in pMMR colorectal cancer
Source: Front Immunol. 2026 May 7;17:1798858. doi: 10.3389/fimmu.2026.1798858 (PMC13189948; doi:10.3389/fimmu.2026.1798858)
Supplement: Supplementary file 3 [file Table3.docx]

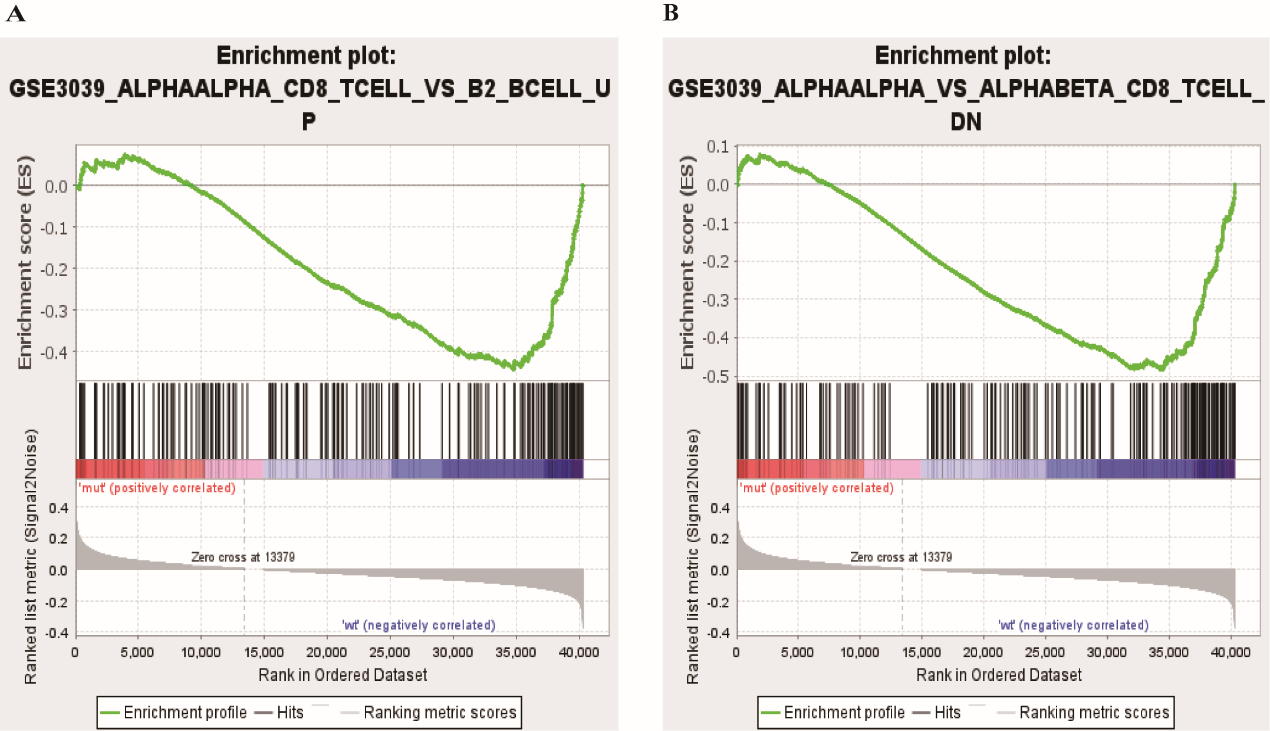


Figure S1. CD8⁺ T Cell Function-Related Gene Expression Profiled by GSEA in TCGA Microsatellite Stable Colorectal Cancer. A: CD8⁺ T cell vs B2 B cell upregulated gene set；B. ALPHAALPHA vs ALPHABETA CD8⁺ T cell downregulated gene set.


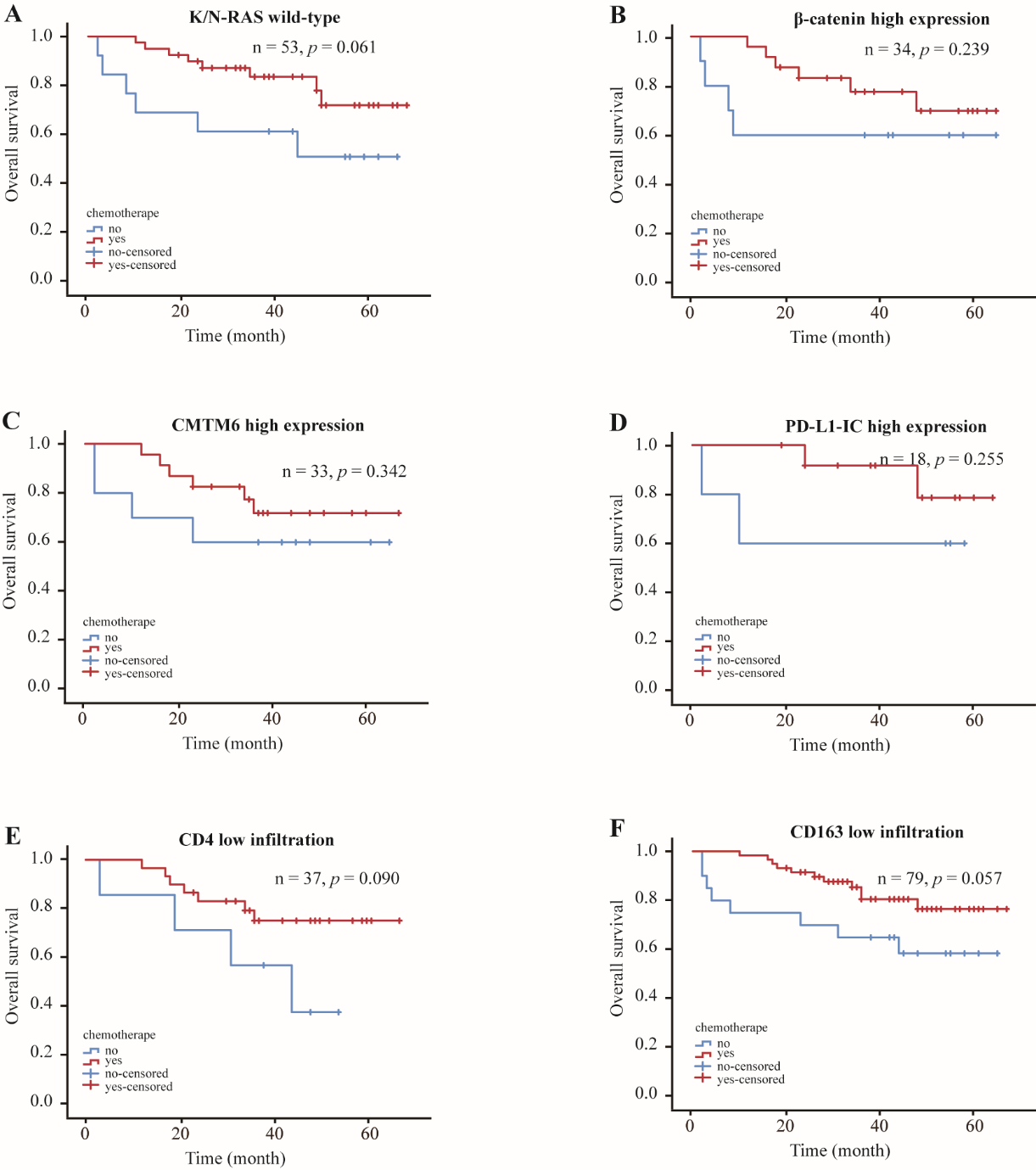


**Figure S2.** Analysis of the effect of chemotherapy on the survival time of TNM stage 3+4 pMMR CRC patients. A. 53 cases with K/N-RAS wild-type; B. 34 cases with β-catenin high-expression; C. 33 cases with CMTM6 high-expression; D. 18 cases with PD-L1-IC high-expression; E. 37 cases with CD4-positive T cell low-infiltration; F. 79 cases with CD163-positive macrophage low-infiltration


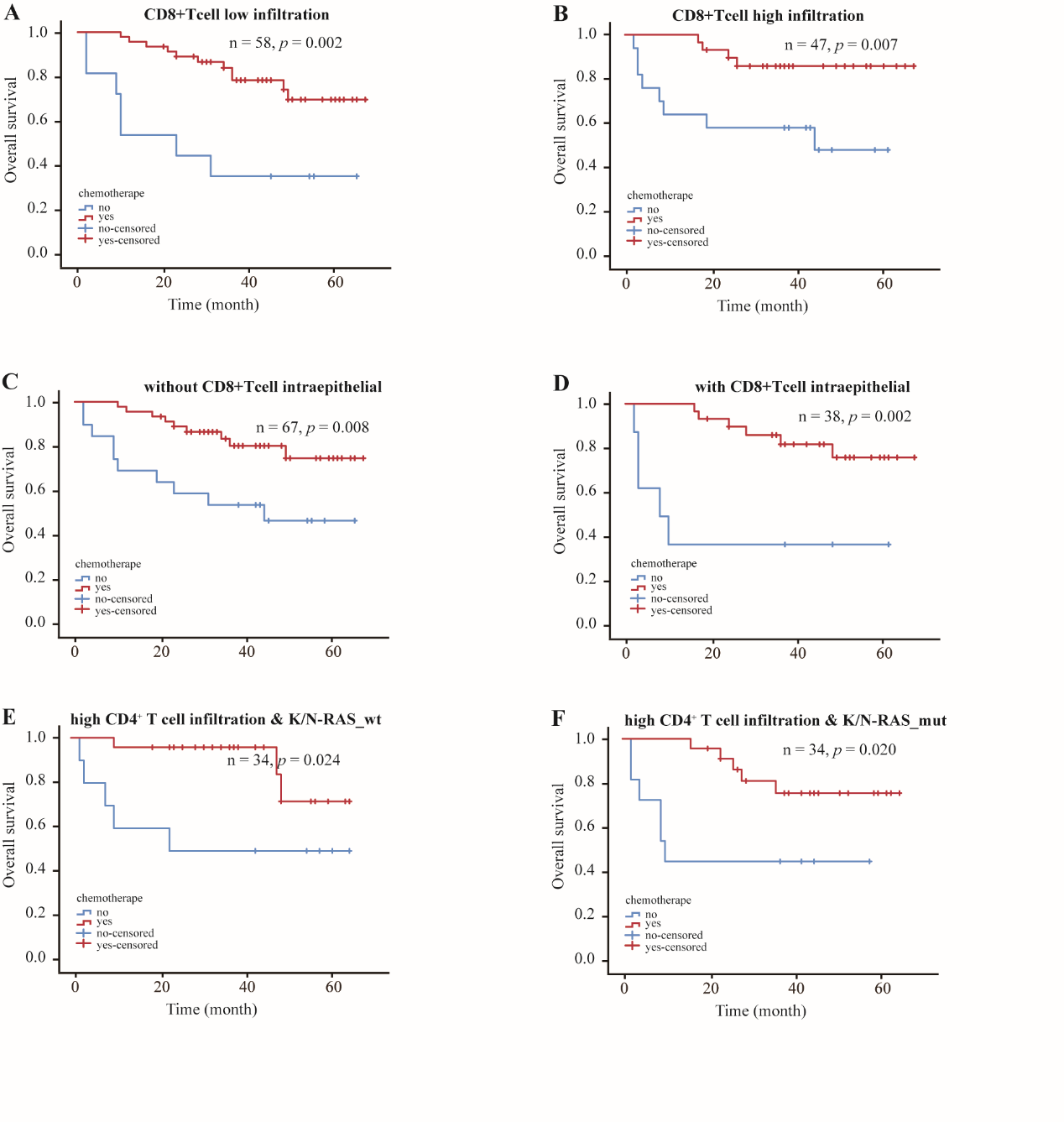


**Figure S3.** Analysis of the effect of chemotherapy on the survival time of TNM stage 3+4 pMMR CRC patients. A. 58 cases with low CD8 positive T cell infiltration; B. 47 cases with high CD8 positive T cell infiltration; C. 67 cases without CD8 positive T cell infiltration into tumor epithelium; D. 38 cases with CD8 positive T cell infiltration into tumor epithelium. E. 34 cases with high CD4 positive cell infiltration and K/N-RAS wild-type; F. 34 cases with high CD4 positive cell infiltration and K/N-RAS mutant-type
